# A novel framework for production planning and class-based storage location assignment: Multi-criteria classification approach

**DOI:** 10.1016/j.heliyon.2024.e37351

**Published:** 2024-09-03

**Authors:** Mehmet Akif Yerlikaya, Feyzan Arıkan

**Affiliations:** a Department of Industrial Engineering, Bitlis Eren University, Bitlis, 13000, Turkey; b Department of Industrial Engineering, Gazi University, Ankara, 06500, Turkey

**Keywords:** Class-based storage location assignment, Production planning, COI, TOPSIS, Decision support system flow

## Abstract

Efficient warehouse management is essential for optimizing inventory, minimizing transportation costs, and enhancing overall performance. This research introduces a novel Mixed-Integer Nonlinear Programming (MINLP) model to address the Storage Location Assignment Problem (SLAP) in warehouse management. Integrating multi-criteria decision-making with strategic production planning, our model advances warehouse operations by allocating storage locations to products strategically, focusing on reducing transportation distances and maximizing storage efficiency. The distinctive innovation of this study is the nuanced application of the Technique for Order Preference by Similarity to Ideal Solution (TOPSIS) results to strategic storage location assignments, enhancing the model's capability to consider a comprehensive evaluation of inventory attributes, including physical characteristics and perishability. This approach evolves TOPSIS's application in warehouse management, enabling it to consider both physical characteristics and perishability of products. The outcomes of TOPSIS, including product classifications and preferences, serve as vital inputs to the mathematical model, facilitating a comprehensive evaluation of storage locations that encompasses spatial, demand-related, and physical aspects of inventory. Additionally, our research introduces a versatile decision support system, adaptable to various operational requirements. This system enhances practical decision-making in warehouse management, accommodating scenarios based on single or multiple criteria, including the cube-per-order index (COI). The research results highlight the significant impact of this innovative approach in enhancing warehouse management. By addressing the complexities of storage location assignment and integrating multiple criteria, we achieve more efficient and cost-effective warehouse operations. The approach has been shown to be adaptable and practical, making it a valuable contribution to the field of logistics and warehouse management.

## 1 Introduction

Effective warehouse management requires consideration of various criteria, including inventory management, storage/unloading/transportation, transportation distance, usage area, and delivery times [1]. Among the pivotal operations within a warehouse, order picking holds a critical role where products are selected from shelves according to customer requirements to assemble orders. Given the substantial volume of order picking processes, optimizing warehouse design and operational criteria becomes paramount for enhancing order picking performance and curbing costs. Therefore, numerous studies in the field of warehouse management focus on the optimization of the order picking process, encompassing activities such as order determination, product picking, order classification, and packaging, all preceding the shipment of items to customers. Warehouse administrators employ diverse controls, encompassing capacity management in picking-classification operations and storage policies, to enhance the responsiveness of their systems. One strategic decision of utmost importance within warehouse operations management revolves around selecting an appropriate order picking method or policy, as this decision significantly influences warehouse design and operations, far more than other decisions. A pivotal decision problem within warehouse operations is the assignment of storage locations. This problem revolves around determining the optimal allocation of storage locations based on specific criteria, with the primary objectives being to minimize transportation time or distance during the storage process and reduce the required space. The proper placement of products in storage locations becomes indispensable before they can be picked to fulfill customer orders. The storage location assignment process entails a set of rules used to allocate products to specific storage locations, thereby establishing the framework for determining the pick activities within the storage system [2].

The literature underscores the pivotal role of storage location assignment and the selection of order picking policies within order picking systems. The class-based policies that utilize the COI technique in classifying products stand out as a crucial tool for evaluating the effectiveness of these policies. However, in warehouses where multiple criteria, such as space, size, cost, weight, and demand, are considered, conflicts among these criteria may arise, making the selection of the best products a challenging problem. In such instances, the SLAP metamorphoses into a MCDM problem [3]. As the field of warehouse management continually evolves and novel technologies are introduced, there is a growing demand for research that addresses the distinct challenges and opportunities presented in this domain [4].

This study introduces an innovative framework that integrates production planning with multi-criteria storage location assignment. Unlike previous research, which often isolated these two aspects, our approach takes a holistic perspective, recognizing their interdependence. We utilize a mixed-integer nonlinear mathematical model that incorporates the TOPSIS class-based policy. This model not only addresses the assignment of products to storage areas but also seamlessly integrates production planning considerations. By concurrently tackling both dimensions, our framework offers a comprehensive solution that effectively handles the complexities of real-world warehouse management scenarios. Additionally, we introduce the TOPSIS class-based policy for storage systems, which encompasses multiple criteria, including COI, weight, and shelf life. This enhances the applicability and effectiveness of the TOPSIS approach in warehouse management. Our research culminates in the development of a decision support system that integrates our mathematical model with various product categorization and assignment methods. This decision support system accommodates a wide range of decision-making scenarios, whether based on single criteria, multiple criteria, or COI index criteria. By providing flexibility and practicality, our decision support system sets itself apart from previous studies that primarily focus on theoretical models.

In terms of the additional value it contributes to the field, our research addresses a critical void in the existing body of knowledge. By considering the integration of product assignment and production planning, we furnish a comprehensive solution that enriches the holistic comprehension of warehouse management. The proposed mathematical model and decision support system supply practical tools for optimizing storage area assignments while factoring in multiple criteria. This leads to heightened efficiency and cost reduction in warehouse operations as our model strategically optimizes product placement. By integrating multi-criteria decision-making, including weight, shelf life, and COI, we ensure optimal utilization of warehouse space and resources. This comprehensive approach significantly reduces operational costs and enhances overall productivity, demonstrating the effectiveness of our model in diverse warehouse environments. This outcome is primarily due to the strategic integration of diverse criteria in our model, specifically weight and shelf life alongside COI, which streamlines the decision-making process. By efficiently allocating storage locations based on these comprehensive factors, our approach minimizes unnecessary handling and space usage, directly contributing to operational cost savings and enhanced productivity in warehouse management. Furthermore, our incorporation of the TOPSIS class-based policy, which extends criteria to encompass weight and shelf life, alongside COI, adds a novel dimension to extant methodologies for managing storage systems. The reason for choosing the shelf life criterion is due to the critical importance of shelf life in the management of perishable products, which is a common challenge in the food industry. Shelf life significantly affects inventory management and decision-making in warehouses, especially when it comes to perishable products. Products with limited shelf life require more strategic planning and efficient allocation to minimize waste and ensure timely distribution. By contemplating multiple factors, our approach empowers warehouse managers to render more informed decisions and attain superior outcomes. To corroborate the efficacy and applicability of our proposed approach, forthcoming research should entail experimental studies conducted within real-world settings. By executing experiments and juxtaposing results against alternative approaches, researchers can further substantiate the efficacy and applicability of our integrated model and decision support system.

The article is structured as follows: Section 2 presents the literature review, followed by Section 3 where the proposed model is introduced. Subsequently, Section 4 elaborates on the core solution approach. Finally, the results and overall evaluation of the paper are presented in Section 5.

## 2 Literature research

The existing literature encompasses a wide range of studies examining warehousing, order picking, warehouse operations, and SLAP. These studies emphasize the significance of optimizing various performance measures within real-world industrial contexts. One specific area of focus is SLAP, which has attracted considerable attention in recent research efforts aimed at optimizing factors such as travel time and space cost [4].

Different storage location assignment policies can be implemented in an order picking system. Turner [5] defines seven storage location assignment policies: class-based, turnover-based, volume-based, dedicated, random, shared storage, and duration of stay policies. The class-based policy, one of the policies under consideration, has had an impact on the process of creating product classes based on various criteria and its practical suitability for real-world applications. In this study, the literature review was conducted under three headings: Studies on class-based storage location assignment, studies on integrated storage location assignment and production planning, and studies on storage location assignment using the MCDM method.

### 2.1 Studies on class-based storage location assignment

Hausman et al. [6] conducted a study focusing on pallet assignment in a warehouse with the objective of minimizing crane transportation time in automatic storage systems. Their research demonstrated the superiority of the class-based storage location assignment policy over percentage-based and random storage policies. Berg [7] addressed the problem of class-based storage location assignment in single command storage and unloading systems. He proposed an efficient dynamic programming algorithm to minimize the average single command cycle time by optimizing class assignment. Le-Duc and De Koster [8] proposed a class-based probabilistic model to estimate the average transport distance of a picking tour. Muppani and Adil [9] employed the COI technique to form product classes for storage location assignment and presented a nonlinear integer programming model that considered transportation costs, storage activities, and space utilization. Li et al. [10] developed a multi-objective mathematical model for SLAP, incorporating order frequency and shelf balance in their classification strategy. Meghelli and Sari [11] proposed a demand-based class-based storage location assignment method for warehouse layout design. Kovacs [4] developed a mathematical mixed-integer programming model for class-based storage location assignment in a warehouse with planned routes, multiple routes, and a multi-command milk-run system. Ene and Öztürk [12] aimed to optimize storage location assignment and order picking system design using stochastic optimization and mathematical modeling techniques. They proposed a class-based integer linear programming model to minimize transport distance. Yang and Nguyen [13] introduced a novel approach based on constrained clustering. Their method takes into account the specific constraints and requirements of the stuck operation. By considering these factors, the proposed approach offers a tailored solution for effectively grouping stuck items in a class-based warehouse. Deng et al. [14] studied the performance of shuttle-based compact storage systems under different storage policies. They used a probability and queueing-based approach to analyze the systems and found that a class-based storage policy with a steep ABC curve and skewed demand rate distribution provided the best performance. Xu and Ren [15] propose a new storage location assignment strategy called multi-pickers-based scattered storage location assignment (MPSSLA) for multi-pickers picker-to-parts picking systems. This strategy allows the same items to be stored in multiple locations, reduces congestion, and improves picking efficiency.

### 2.2 Studies on integrated storage location assignment and production planning

Wilson's [16] pioneering academic study integrated the optimization of reorder points and storage location assignment in a mathematical model. The objective was to minimize order picking costs and inventory costs by considering various cost factors such as production, inventory, setup, and transportation costs. Daellenbach [17] focused specifically on SLAP and incorporated inventory planning and renewal policies into the analysis. Hodgson and Lowe [18] addressed the challenge of integrated product assignment and reorder point determination. Malmborg and Deutsch [19] formulated a cost model that captured the relationship between inventory costs and order picking costs, taking transportation costs into account as well. Their contributions advanced the understanding and optimization of storage systems. Malmborg [20] developed a successful model for evaluating alternative storage location assignment policies based on order picking costs, considering total space allocation and storage location. Kültürel et al. [21] combined SLAP with the continuous review inventory control model (Q, r). Hassini [22] focused on storage assignment and renewal times, particularly in situations where the number of products is lower than the number of available storage areas. Zhang et al. [23] proposed an integrated strategy that merged SLAP with the capacitated lot size problem commonly found in the literature. They developed a mixed-integer linear programming model to minimize the overall cost of production and warehouse operations by considering the multi-product capacitated lot size problem and the dynamic SLAP. In their study, Yerlikaya and Arikan [24] tackled the complex problem of class-based storage location assignment and production planning. They used a Mixed Integer Nonlinear Program (MINLP) formulation with the goal of improving efficiency and reducing operational costs, particularly by optimizing the allocation of products to storage areas and integrating these decisions with production planning. The results of the study demonstrate that the proposed model outperforms the existing solutions in the literature, leading to cost reduction, location savings, and improvements.

### 2.3 Studies on storage location assignment using the MCDM

MCDM methods have proven to be effective and valuable in addressing the complexities of storage systems that involve conflicting criteria. Fontana and Calvante [25] applied the ELECTRE TRI method to evaluate and rank products in warehouse environments. They focused on stock strategies, product characteristics, and demand as their primary criteria. While their objective included assessing products based on specific attributes such as quality or suitability, it's crucial to note that their evaluation was not limited to these aspects. Instead, they employed a more comprehensive approach, considering a combination of factors like stock strategy, product characteristics, and demand to rank products and allocate them to suitable storage locations. This methodology was aimed at aligning product allocation with decision-makers’ preferences, reflecting a broader spectrum of criteria than just quality or suitability alone. Similarly, Fontana and Cavalcante [1] employed the PROMETHEE method to solve the SLAP based on a class-based policy and decision maker's preferences. In a different approach, Da Silva et al. [26] proposed the SMARTER & lexicographic method as an alternative to COI for product sorting in dedicated policies. Fontana and Nepomuceno [27] developed a multi-criteria decision model that was custom-designed for the classification of products in high-level storage systems, such as multi-layer warehouses. Their model's primary objective was to tackle the Storage Location Assignment Problem (SLAP) by taking various criteria into consideration. In a similar vein, Micale et al. [28] proposed a hybrid approach to address uncertainty. They combined the ELECTRE TRI and TOPSIS methods to handle this challenge. Initially, they used the ELECTRE TRI method to allocate Stock Keeping Units (SKUs) to different rack levels. Subsequently, the TOPSIS method was employed to pinpoint precise storage locations within each level for the placement of SKUs. The study conducted by Yerlikaya [29] proposes the utilization of the fuzzy PROMETHEE (F-PROMETHEE) method for ranking products and assigning them to the most suitable storage locations in warehouse systems with uncertain demands. This approach allowed for more robust decision-making in the face of uncertainty. These studies highlight the versatility and applicability of MCDM methods in addressing the complexities of SLAP, offering insights into different methodologies and approaches to optimize storage systems. Stevic et al. [30] propose a fuzzy multicriteria decision-making model for evaluating information technologies in warehouse order picking. The model integrates IMF SWARA-Z and fuzzy EDAS-Z methods based on Z numbers. They evaluate barcode, pick-to-light, pick-to-voice, and pick-to-vision technologies and determine pick-to-vision as the best option. Tian et al. [31] have developed a mathematical model based on the bi-population differential artificial bee colony (BDABC) for production planning, considering the energy efficiency of a dynamic flexible job shop with a wide variety of small batch jobs. This model aims to minimize the total energy consumption (TEC), make span, and machining cost. Alqahtani [32] has focused on the storage allocation problem to determine the optimal inventory levels of Stock-Keeping Units (SKUs) and to place SKUs in the most efficient locations. The study describes a hierarchical top-down technique to merge separate decision-making processes. In this example, using the proposed method, the Analytic Hierarchy Process (AHP) is shown to provide valuable insights for solving the case study at hand. Additionally, an alternative rack system named 'Radio Shuttle,' which has received the highest evaluation and offers the shortest product retrieval time, is recommended.

While the existing literature has limited studies on Storage Location Assignment Problem (SLAP) utilizing Multi-Criteria Decision-Making (MCDM) methods, it's noteworthy that these studies typically focus solely on product assignment according to a specific MCDM method. Moreover, they often overlook the crucial aspect of production planning. In contrast, our study introduces an integrated mathematical model that bridges this gap by effectively addressing two fundamental dimensions: the allocation of products to storage areas and the strategic planning of production activities. The allocation of products to storage areas and the strategic planning of production activities in our model involve synchronizing warehouse operations with overall production goals. In our research, we not only propose an integrated mathematical model for the Storage Location Assignment Problem (SLAP) but also explicitly demonstrate the model's capacity to synchronize warehouse operations with production planning. Specifically, the methodology section details the algorithms and decision-making processes that ensure optimal product placement is not considered in isolation but is intricately linked with production schedules, demand forecasts, and resource availability. This approach is further validated in the results section, where we present case studies and simulations that exemplify how our model achieves this integration, leading to more efficient and responsive warehouse management.

Our approach incorporates a class-based policy, which leverages the TOPSIS method for creating product classes within storage systems. This strategy takes into account multiple essential criteria that influence decision-making. The primary goal of this comprehensive model is to boost the efficiency of both storage allocation and production planning, thus providing a more holistic approach to the Storage Location Assignment Problem. For a comparative analysis of our proposed model with previous research employing multi-criteria methodologies, please refer to Table 1.

**Table 1:** Studies using the multi-criteria approach.

Study	Year	Method	Criteria	Production Planning	Decision Support System
Fontana and Calvante [25]	2010	ELECTRE TRI	Quality, Suitability, Stock Strategy, Product Characteristics, Demand	No	No
Fontana and Cavalcante [1]	2012	PROMETHEE	COI, product characteristics	No	No
Da Silva et al. [26]	2014	SMARTER & Lexicographic	COI	No	No
Fontana and Nepomuceno [27]	2015	TOPSIS	COI, product characteristics	No	No
Micale et al. [28]	2016	ELECTRE TRI and TOPSIS	COI, order frequency	No	No
Yerlikaya [29]	2017	Fuzzy PROMETHEE	COI, order frequency	No	No
Stevic et al. [30]	2018	IMF SWARA-Z and fuzzy EDAS-Z	–	No	No
Tian et al. [31]	2023	BDABC & Mathematical Model	(TEC), Makespan, Machining Cost	Yes	No
Alqahtani [32]	2023	ABC & AHP	Cost, utilization, load access, and stock cycle speed	No	Yes
This Study	–	TOPSIS & Mathematical Model	COI, weight, Shelf life	Yes	Yes

When examining existing literature on the SLAP, traditional methods often focus on factors such as order quantity and order frequency. In addition, they implicitly consider the relationship between demands, which is understanding how various products' demands influence each other within a warehouse setting. This involves recognizing patterns like some products being frequently ordered together or affecting each other's sales. These studies aim to minimize basic criteria such as space, distance, picking time, order picking effort, and associated costs while determining the location of products in the storage. However, our proposed model brings significant innovations to the field, as outlined below:

• Integration of Multiple Criteria: Unlike traditional approaches that focus on a limited set of criteria, our model incorporates multiple criteria into the product assignment process. We consider factors such as weight and shelf life, in addition to the widely used COI which primarily focuses on space, demand, and stock level criteria. By integrating these dimensions, our approach encompasses a comprehensive set of factors crucial for efficient storage location assignment. This multi-criteria approach allows for a more holistic evaluation of products, leading to improved decision-making in warehouse management.
• Application of MCDM Method: Our model utilizes the Technique for Order Preference by Similarity to Ideal Solution (TOPSIS), which is a well-established Multiple Criteria Decision Making (MCDM) method. This approach enables the evaluation of products based on various criteria simultaneously, providing a more comprehensive assessment than single-criterion models commonly found in the literature.
• Development of a Decision Support System: One of the key innovations of our approach is the creation of a decision support system that assists decision-makers in choosing the most suitable product assignment policy. Unlike previous methods that often consider only a single criterion or a limited set of criteria for class formation, our system accommodates multiple criteria. This flexibility enhances its practicality and utility in real-world warehouse management scenarios.
• Class-Based Policy: Our model's 'Class-Based Policy' for product assignment involves grouping products into distinct classes based on their unique characteristics and criteria. This classification is not arbitrary; it is based on specific attributes such as weight, shelf life, demand patterns, and other relevant factors that impact storage and retrieval efficiency. By grouping similar items together, the policy allows for more tailored and efficient storage location assignment. The benefits of this class-based approach are twofold. Firstly, it optimizes the use of warehouse space. Products with similar storage and access requirements are grouped together, reducing the need for excessive movement within the warehouse and thus maximizing space utilization. Secondly, it reduces transportation costs. To substantiate our strategy's efficiency, the reduction in picking distances achieved through class-based product placement is quantitatively analyzed and evidenced in the results section, showcasing improved order fulfillment speeds and lowered operational costs. This class-based policy is designed to enhance the overall efficiency of warehouse operations by considering the unique attributes of each product, thereby facilitating more effective storage management and cost savings.

In summary, our proposed model offers a comprehensive and innovative approach to storage location assignment in warehouse management. By considering multiple criteria, applying MCDM methodology, developing a decision support system, and implementing a class-based policy, our model addresses the complexities of real-world warehouse operations more effectively than traditional methods. This innovation has the potential to enhance order picking efficiency, reduce costs, and improve overall warehouse space utilization, making it a valuable contribution to the field of warehouse management.

## 3 Problem definition and formulation

The primary challenge in practical storage systems arises when the available space in the warehouse proves insufficient during the transfer of products from the production area. The problem at hand revolves around the layout, handling, storage, and production processes within the warehouse. In this scenario, products produced in the production area undergo classification, placement in suitable storage locations, and subsequent transportation to the assigned delivery point for customer orders. The objective is to assign product classes to the most appropriate storage locations, thereby minimizing costs associated with transportation, storage, production, inventory, and setup. The product flow process within the warehouse can be outlined as follows:

• Manufactured products originate from the production area.
• These products are gathered from the production area and conveyed to storage locations, where they are placed.
• Forklift trucks are utilized to collect the products from these locations and assign them to their respective storage locations.
• Subsequently, forklift trucks transport the products from these locations to the I/O point.

**Fig. 1:**
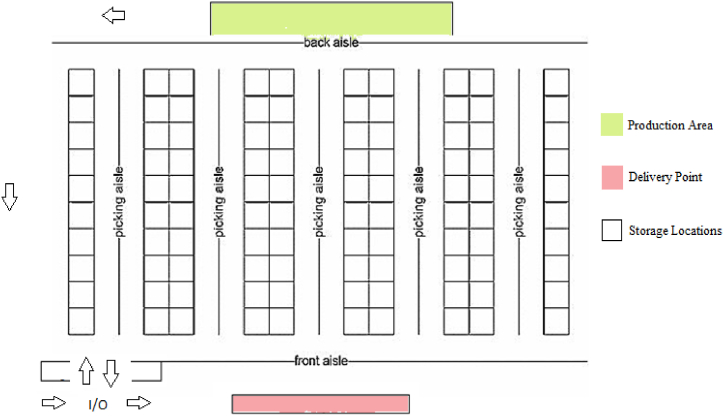
A sample warehouse layout for the problem.

A sample warehouse layout for the problem is given in Fig. 1. The warehouse system discussed in the sample consists of the production area, delivery point to the customer, storage locations, and an input-output door (I/O). According to the description, the sample warehouse layout includes the following components:

Production Area: This is the area where goods or products are manufactured or processed.

Delivery Point: This is the location within the warehouse where goods are prepared for shipment to customers.

Storage Locations: These are storage systems consisting of vertical frames and horizontal beams that are used to store palletized goods. Pallet racks allow for efficient use of vertical space and easy access to stored items.

I/O: This refers to the door or entrance through which goods enter or exit the warehouse. It serves as a point of transfer for incoming and outgoing shipments.

Without further details or a visual representation, it's difficult to provide a more detailed description of the warehouse layout. The costs related to the transportation of products from the production area to the storage locations and from the storage locations to the I/O point were determined according to the operator movement. The cost of moving to two different points in warehouses with a single I/O point is not the same due to the difference in distance. The problem is based on the following assumptions:

• A class-based storage location assignment policy is employed.
• All products are transported and stored on pallets.
• Pallets vary in size and weight, but their impact on transportation costs is disregarded.
• A specific number of storage locations are utilized.
• Products are moved from the production area to storage locations and from storage locations to the delivery point using a forklift with single command capability.
• The system includes an input/output (I/O) point and a production area where products originate.
• Costs associated with placing and retrieving products are directly proportional to the distance traveled.
• Demand is known, and stockouts are not permitted.
• Each product has a defined shelf life, and expired products are transported from storage locations to the I/O point.
• Setup costs are determined by the time required for manufacturing a product within the production area.

In the domain of storage location assignment, various methods are employed for class formation and allocation in systems where multiple criteria are crucial. One of the widely adopted techniques in the literature is the COI technique. The COI technique, three criteria, namely space, demand, and stock level are taken into account. However, in storage systems, when various criteria, including space, cost, and demand, are considered alongside different factors, the situation changes. For instance, when a product's shelf life is over, it is collected regardless of whether there is demand for it. This means that these products can be retrieved not only in response to immediate demand but also in specific situations related to their shelf life. In this study, a nonlinear mathematical model has been developed for the integrated production planning and multi-criteria SLAP, and the TOPSIS method, which allows the evaluation and classification of products according to multiple criteria has been preferred. The reason why the TOPSIS method is preferred is that, in the proposed model's warehouse system, products (alternatives) cannot be used interchangeably. In other words, alternative products are dissimilar, and the criteria considered for evaluation are different. In this complex situation, the TOPSIS method identifies the most suitable options, taking into account the dissimilarity of products and diverse evaluation criteria, ensuring that the selected alternative is the most appropriate for the specific context. The combination of TOPSIS and COI methods is clearly evident in the decision-making process of this study. The COI method is initially used to classify and position products based on their demand, space requirements, and stock levels. The results obtained from the COI method, including product classification and COI values, serve as inputs for the TOPSIS method. The TOPSIS method in our model further scrutinizes and ranks the products by considering additional criteria such as weight and shelf life, alongside the already integrated COI. This scrutiny involves assigning numerical values to each product based on these criteria and determining their relative performance. The ideal distance values (C*) obtained from TOPSIS play a crucial role in establishing the final placement of products within the warehouse.

The application steps of the proposed mathematical model are as follows:

1. Determining the criteria for the products to be classified (If the criteria are space, demand, and stock level, the COI index is used),
2. Creation of the decision matrix,
3. Weighting the criteria,
4. Determining the C* value for each product with the TOPSIS method,
5. Adding the C* value to the proposed base model as a class formation priority constraint,
6. Solution of the mathematical model.

### 3.1 Decision support system flow

**Fig. 2:**
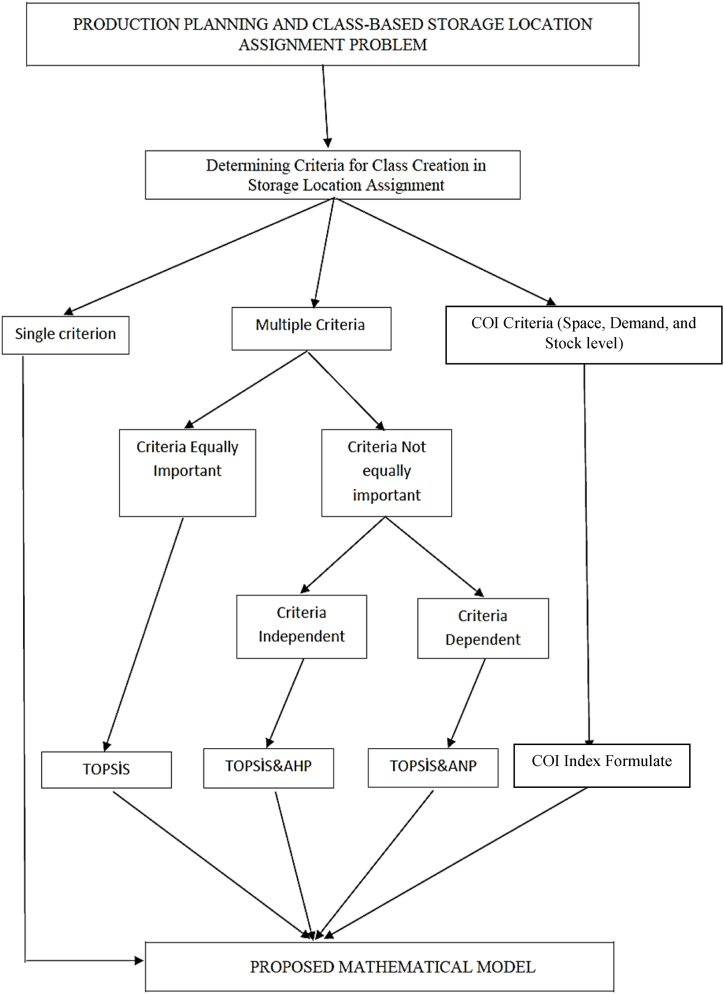
Decision support system flow.

In the proposed model, class formation can be determined by various methods depending on the storage system's structure. Accordingly, our decision support system flowchart, as illustrated in Fig. 2, assists decision-makers in selecting the most appropriate method for their specific needs. This selection can be based on a single criterion, such as Cost, Demand, or Space, a combination of multiple criteria like TOPSIS, or COI criterion. Each of these options offers a different approach to class formation, with COI particularly focusing on space utilization and demand patterns in the warehouse. This inclusion ensures a comprehensive and flexible system that can adapt to various operational requirements and priorities in storage management.

In the decision support system flow, if the creation of the product class depends on a single criterion, the basic mathematical model that does not contain any prioritization constraints is solved and the products are classified according to the assignments that minimize the order picking cost. The only criterion here is the cost defined by the objective function. The specifics of this function, including the formula used to calculate cost, are detailed in a subsequent section of this paper. If the creation of the product class depends on multiple criteria, the C* values are determined for each product with the TOPSIS method, these C* values are added to the basic mathematical model as a class formation priority constraint and solved as a new variation of the proposed basic mathematical model with priority constraints added. In the adapted mathematical model for creating product classes based on multiple criteria, when the criteria are not equally important, the weights of these criteria are determined using AHP or, depending on the interdependencies of the criteria, the Analytic Network Process (ANP) method. The resultant criterion weights are then utilized as inputs for the TOPSIS method. "In the literature, the most frequently used method for creating product classes, known as the Cost of Item (COI) method, typically considers three criteria: space, demand, and stock level. This approach is particularly favored in warehouse systems where efficient space utilization is crucial. When the COI method is chosen for product class creation in alignment with the system's requirements, a COI value can be determined for each product. These values can be incorporated into the mathematical model as constraints for class formation priority, allowing for a variation of the proposed mathematical model designed to accommodate multiple criteria in product class creation.

Regarding Fig. 2, it is evident that the choice between using the COI index method or a multi-criteria approach depends on the specific criteria under consideration. If the criteria include space, demand, and stock level the COI index method is employed. However, if the criteria differ from these or if more than two criteria are involved, a multi-criteria approach is implemented.

### 3.2 TOPSIS method

For storage assignment, many methods are used for class formation and allocation in storage systems where multiple criteria are important. One of these methods is the most widely used COI technique. The COI technique is especially used in warehouses where the space usage is not identical and the rack columns are parallel to the I/O point. However, this situation changes in storage systems where different criteria are taken into account as well as criteria such as space, stock level, cost, and demand. In this study, the TOPSIS method, which allows the evaluation and classification of products according to multiple criteria, was preferred for storage assignment. It's important to note that while TOPSIS has been used in previous literature for storage assignment, it had not been employed as an input in any mathematical model of this nature before. TOPSIS has the ability to work with different scales and values among criteria. Hence, in situations where products in the warehouse exhibit variations in measurement units (such as weight, shelf life, stock level) and encompass diverse value ranges, the TOPSIS method is specifically favored. Unlike some other Multiple Criteria Decision Making (MCDM) methods, TOPSIS excels in its ability to normalize these varying scales, making it a preferred choice. The TOPSIS method solution process consists of 6 steps [33]:

1) Formulating the Decision Matrix (A): The decision matrix consists of alternatives represented in the rows and the decisive criteria for decision-making represented in the columns. This decision matrix is formulated as in Equation (1).

(1) $$A=\left[\begin{array}{cccc} a_{11} & a_{12} & ... & a_{1n}\\ a_{21} & a_{22} & ... & a_{2n}\\ . & & & .\\ . & & & .\\ . & & & .\\ a_{m1} & a_{m2} & ... & a_{mn} \end{array}\right]={\left[a_{ij}\right]}_{mxn}$$

2) Constructing the Normalized Decision Matrix (R_ij_): The normalized decision matrix is obtained by normalizing the values in the decision matrix using Equation (2).

(2) $$r_{ij}=\frac{a_{ij}}{\sqrt{\sum_{k=1}^{m}a_{kj}^{2}}}$$

3) Determining the Weighted Normalized Decision Matrix (v_ij_): Each element of the normalized decision matrix is multiplied by the corresponding weight value (w_i_) assigned to the criterion, resulting in a weighted normalized decision matrix. In this study, equal weights are assigned to all criteria.
4) Establishing the Positive Ideal Solution (V^+^) and Negative Ideal Solution (V^−^): The TOPSIS methodology is designed to identify alternatives that are closest to the positive ideal solution and furthest from the negative ideal solution by using Euclidean distance. This is achieved by calculating the relative proximity of each alternative. In reference to the Equation (3) and Equation (4), the sets J and J′ are defined, where J represents the set of criteria associated with maximizing objectives, and J′ is the set of criteria associated with minimizing objectives. The Negative Ideal Solution (V^−^) can be defined as the vector of worst performance values for each criterion. On the other hand, the Positive Ideal Solution (V^+^) is defined as the vector of best performance values for each criterion.

(3) $$V^{+}=\left\{\left(\max_{i}v_{ij}|j\in J\right),(\min_{i}v_{ij}|j\in J^{\prime }\right\}$$

(4) $$V^{-}=\left\{\left(\min_{i}v_{ij}|j\in J\right),(\max_{i}v_{ij}|j\in J^{\prime }\right\}$$

5) Computing Discrimination Measures: The TOPSIS method employs dimensional Euclidean measurement to quantify the deviations of each alternative's criterion values from the positive ideal (S_i_^+^) and negative ideal (S_i_^−^) solution sets, thereby determining their discrimination measures. The v_j_^+^ and v_j_^−^ values, introduced in Equations (5), (6)), represent the positive and negative ideal solutions for each criterion, respectively. These solutions are determined based on the best and worst values associated with each criterion.

(5) $$S_{i}^{+}=\sqrt{\sum_{j=1}^{n}{\left(v_{ij}-v_{j}^{+}\right)}^{2}}$$

(6) $$S_{i}^{-}=\sqrt{\sum_{j=1}^{n}{\left(v_{ij}-v_{j}^{-}\right)}^{2}}$$

6) Calculation of The Ideal Distance Values to Ideal Solution (C_i_*): The relative proximity (C_i_*) of each alternative to the ideal solution is determined by evaluating the separation measures between the positive ideal and negative ideal solutions, Equation (7). C_i_* represents the ratio of the discrimination measure related to the negative ideal solution to the total discrimination measure. The value of C_i_* ranges from 0 to 1, where C_i_* = 1 indicates the alternative's absolute proximity to the positive ideal solution, while C_i_* = 0 implies its proximity to the negative ideal solution.

(7) $$C_{i}^{*}=\frac{S_{i}^{-}}{S_{i}^{-}+S_{i}^{+}}$$

### 3.3 Notations

The proposed model encompasses various parameters including the number of storage locations, products, forecasted demand, planning horizon, and costs associated with production, travel, storage, setup, and reservation. The notations used in the model are defined as follows:

*Indices:*

i: Index representing a specific product (i = 1, 2, ..., N)

c: Index representing a class (c = 1, 2, ..., C = N)

t: Index representing a period within the planning horizon (t = 1, 2, ..., T)

l: Index representing a storage location (l = 1, 2, ..., L)

*Variables:*

x_it_ : Quantity of product i produced during period t.

s_it_ : Inventory level for product i at the end of period t.

y_it_ : Binary variable (1 if product i is produced during period t, 0 otherwise).

q_ic_ : Binary variable (1 if product i is assigned to class c, 0 otherwise).

z_lc_ : Binary variable (1 if class c is assigned to location l for the planning horizon, 0 otherwise).

*Parameters:*

Z: Objective value in terms of currency ($)

P_l_: Unit cost of moving one product from the production area to storage location l

O_l_: Unit cost of moving one product from storage location l to the output point

R_l_: Unit cost of reserving storage location l

h_it_: Unit inventory cost for product i in period t

c_it_: Unit production cost for product i in period t

u_it_: Unit setup cost for product i in period t

d_it_: Demand quantity for product i in period t

v_it_: Capacity for product i in period t

f_t_: The key resource constraint on production, signifying the setup time capacity for the production area.

M: A large scalar value representing an important limiting factor in production

C_i_*: TOPSIS value (The Ideal Distance Values to Ideal Solution)

### 3.4 Model

The proposed integrated problem is formulated as a mixed-integer nonlinear programming (MINLP) model, which aims to minimize the objective function and optimize the decision variables. By incorporating both integer and nonlinear components, the MINLP model provides a comprehensive framework for addressing the complex nature of the problem at hand.

Objective function:

(8) $$\begin{array}{c} Z_{\min}=\sum_{c}\sum_{l}R_{l}.z_{lc}+\sum_{c}\sum_{t}\left(\left\{\frac{\sum_{l}p_{l}∙z_{lc}}{\sum_{l}z_{lc}}\right\}∙\sum_{i}x_{it}.q_{ic}\right)+\sum_{c}\sum_{t}\left(\left\{\frac{\sum_{l}O_{l∙}z_{lc}}{\sum_{l}z_{lc}}\right\}∙\sum_{i}d_{it}∙q_{ic}\right)\\ \sum_{i}\sum_{t}\left(c_{it}∙x_{it}+u_{it∙}y_{it}+h_{it}∙s_{it}\right) \end{array}$$

Constraints:

(9) $$\begin{array}{cc} \sum_{c}z_{lc}\leq 1 & \forall l\text{,} \end{array}$$

(10) $$\begin{array}{cc} \sum_{c}q_{ic}=1 & \forall i\text{,} \end{array}$$

(11) $$\begin{array}{cc} d_{it}\leq x_{it}+s_{it-1} & \forall it\text{,} \end{array}$$

(12) $$s_{it}=x_{it}-d_{it}+s_{it-1}\forall it\text{,}$$

(13) $$\begin{array}{cc} \sum_{i}\left(x_{it}+s_{it-1}\right).q_{ic}\leq \sum_{l}z_{lc} & \forall ct\text{,} \end{array}$$

(14) $$\begin{array}{cc} x_{it}\leq y_{it}.M & \forall it\text{,} \end{array}$$

(15) $$\begin{array}{cc} \sum_{i}x_{it}.v_{it}\leq f_{t} & \forall t\text{,} \end{array}$$

(16) $$\begin{array}{cc} C_{i}^{*}.q_{ic}\geq C_{i^{\prime }}^{*}.q_{i'c'} & \forall i\neq i^{\prime },\forall c<c^{\prime } \end{array}$$

(17) $$\begin{array}{cc} \frac{\sum_{l}\left(P_{l}+O_{l}\right).z_{lc}}{\sum_{l}z_{lc}}\leq \frac{\sum_{l^{ı}}\left(P_{l^{\prime }}+O_{l^{\prime }}\right).z_{l'c'}}{\sum_{l^{ı}}z_{l'c'}} & \forall l\neq l^{\prime }\;\text{and}\;\forall c<c^{\prime }\text{,} \end{array}$$

(18) $$\begin{array}{cc} z_{lc}\in \left\{0,1\right\} & \forall lc\text{,} \end{array}$$

(19) $$\begin{array}{cc} q_{ic}\in \left\{0,1\right\} & \forall ic\text{,} \end{array}$$

(20) $$\begin{array}{cc} x_{it}\geq 0 & \forall it\text{,} \end{array}$$

(21) $$\begin{array}{cc} s_{it}\geq 0 & \forall it\text{,} \end{array}$$

(22) $$\begin{array}{cc} y_{it}\geq 0 & \forall it\text{,} \end{array}$$

The objective function, Equation (8), of the proposed MINLP model encompasses four cost terms: the cost of reserving locations for product classes, travel costs from the production area to storage locations, transportation costs from storage locations to the I/O, and production planning costs. The objective is to assign product classes to optimal locations and optimize production planning to minimize the total cost. Equation (9) ensures that each location is assigned to at most one class, while Equation (10) ensures that each product is assigned to a single class. Classes are determined based on production and demand flow to minimize space and transportation costs. Equation (11) guarantees that the sum of production quantities and inventory levels meets the demand for each product. Equation (12) represents the inventory balance, and Equation (13) restricts the sum of production quantities and inventory levels for products assigned to a class to be within the reserved locations for that class. Equation (14) represents the production limitation for each period. The variable "M" denotes a very large number determined in the context of production capacity and product demand. Equation (15) represents the production capacity limit. Equations (16), (17) in our mathematical model are designed to prioritize the assignment of products within the storage classes based on their TOPSIS scores. Equation (16) is formulated to give priority to products with higher TOPSIS scores to be assigned to a preferred class c, which could be a location closer to the dispatch area to minimize picking time for high-demand or high-priority items. This is achieved by setting a higher ranking or preference for products within this class, ensuring that products that score well on the TOPSIS evaluation are more likely to be placed in these optimal locations. On the other hand, Equation (17) deals with products that have lower TOPSIS scores, assigning them to a different class c', potentially further from the dispatch area. This is suitable for items with less frequency or urgency in order picking. Here, i and i′ represent indices of products, and the constraints work to allocate these products to classes c or c' based on their respective TOPSIS scores. These constraints ensure that the storage location assignment is aligned with the multi-criteria decision-making outcomes derived from the TOPSIS analysis, thereby optimizing the warehouse layout according to the strategic goals of the operation. Equations (18), (19) are binary integer and variable constraints, while Equations (20), (21), (22) enforce positive variable constraints.

## 4 Solution approach

In this section, a solution was presented using GAMS/BARON optimization software to test the validity and solvability of the proposed mathematical model. First of all, the C* of each product were obtained with the TOPSIS method for the classification of products according to multiple criteria and they were utilized as a set of a constraints' input in the proposed mathematical model. The TOPSIS method solution was obtained by the Microsoft Excel program. For the mathematical model solution, a random data set (10-product, 3-period, 30-location) produced by the Microsoft Excel program were used. The example case presented in this article discusses the process of integrating a multi-criteria classification system for warehouse management in a food company using the TOPSIS method and considering class-based production planning. A food company scenario is referenced to provide a practical context for this model. Efficiency and cost reduction are paramount objectives in this approach, responding to the compelling need for companies to remain competitive. Effective warehousing ensures timely order fulfillment and a competitive edge, while cost reduction is fundamental for profitability and resource management. Optimizing warehouse strategies enables streamlined operations, efficient resource allocation, and serves as a valuable reference for similar challenges in logistics and warehouse management.

### 4.1 Prioritizing products with TOPSIS method

The proposed model is for the warehouse of a food company producing dry food, and 4 criteria have been determined for this. These criteria for ranking the products are equally important and are as follows:

• COI Index: The COI method, introduced by Hesket [34], is utilized to determine the ratio between the maximum space required per SKU and the order frequency for product positioning or class formation (Equation (23)). In this equation, D_i_ represents the total demand for product i during period t, f_i_ denotes the space required to stock product i, and I^t^_i_ represents the stock level of product i in period t.

(23) $$COI_{i}=\frac{f_{i}\;x\;\left[I_{i}^{t}\right]}{D_{i}}$$

• Weight (kg): It is the weight of the product. In our model, the 'weight' criterion is accorded significant importance, warranting its maximization in the decision-making process for storage location assignment. Heavier products require more resources to move, so placing them closer to dispatch areas reduces handling efforts. This strategic placement minimizes transport costs and improves safety within the warehouse, justifying the maximization of the weight criterion in storage location assignments.
• Shelf life (year): It is the period during which a product can be stored before the product becomes suitable for consumption or sale, or the period during which a product with a certain expiry date can remain on sale. The inclusion of shelf life as a criterion in our model highlights its relevance and applicability to industries dealing with perishable goods, where efficient and timely allocation of storage space is crucial for maintaining product quality and minimizing losses

The decision matrix, consisting of 10 items based on the provided data, is presented in Table 2. Following the application of the TOPSIS method to this decision matrix, the Ci* values for each product are shown in Table 3. These Ci* values, which determine the proximity of products to the I/O point, indicate that product 5 and product 3 should be positioned closest to the I/O point. The C* values for each product have served as constrained inputs for the integrated production planning and product assignment model, which categorizes products based on multiple criteria.

**Table 2:** Decision matrix.

CRITERIA →	COI_i_			Weight	Shelf Life
	Space (f_i_)	The maximum stock level (I^t^_i_)	Demand Quantities (D_i_)		
ALTERNATIVE↓	MIN	MIN	MAX	MAX	MIN
Product1	2,5	7	7	10	1,5
Product2	1,8	6	5	7	0,9
Product3	2,3	8	6	9	0,6
Product4	3,1	7	4	11	2
Product5	1,6	9	8	12	0,7
Product6	3,6	8	7	8	0,8
Product7	2,1	9	3	10	1,3
Product8	1,2	5	2	9	1,6
Product9	1,8	7	6	11	1,9
Product10	2,6	5	5	10	1,8

**Table 3:** Positive, negative, and ideal distance values.

	S+	S-	C*(i)
Product1	0,110	0,080	0,420
Product2	0,087	0,083	0,488
Product3	0,082	0,113	0,578
Product4	0,138	0,047	0,252
Product5	0,060	0,131	0,686
Product6	0,106	0,093	0,467
Product7	0,125	0,047	0,272
Product8	0,121	0,100	0,452
Product9	0,109	0,080	0,423
Product10	0,121	0,070	0,365

### 4.2 Solution of the mathematical model

The parameters of the sample problem, which involves 10 products, 3 periods, and 30 locations, are summarized in Table 4. Optimal results were obtained for the mathematical model using GAMS/BARON optimization software. The solutions determine the production quantities for each product, the number of production setups based on different batch sizes, the assignment of products to suitable locations based on demand and classification, and the retrieval of products from locations categorized by classes within specific time intervals.

**Table 4:** Datasets of the example problem.

R_L_	10	h_it_	3,02
P_L_	1,5-5,5	d_it_	2–8
O_L_	0,5-4,5	v_it_	1
c_it_	5,82-6,73	f_t_	80
u_it_	15–30	M	20

Table 5 provides a detailed breakdown of production quantities for different products during the months of January, February, and March. It is important to note that these production quantities vary significantly between products and months. For example, in January, Product 1 was produced in a quantity of 3 units, followed by 2 units in February, and 2 units in March. A similar pattern is observed for other products, each with its own unique production quantities for each of the three months. This variance in production quantities allows for a deeper understanding of the dynamics of the production process and its impact on inventory management. Table 6 illustrates the allocation of products to distinct classes, with a total of 4 classes. Within these classes, the distribution of products is as follows: Class 1 includes 2 products, Class 2 contains 5 products, Class 3 encompasses 1 product, and Class 4 consists of 2 products. Each product is assigned to a specific class, which influences its handling and storage. For instance, Product 1 is categorized under Class 2, Product 2 also belongs to Class 2, Product 3 is associated with Class 1, and Product 4 falls into Class 4. While the class assignments are provided, the exact count of products in each class is now specified in this table. Table 7 displays the arrangement of storage locations according to different classes. Each class is associated with specific locations. For instance, Class 1 products are stored in Locations 1 to 6. The location assignments for other classes are provided in the table. Table 8 presents the numerical results obtained from the optimization of the sample problem. The "Object Value" column represents the value of the optimized objective function, which reflects the problem's objective. The GAP value calculated by the GAMS program during the solution process indicates how much the optimized value deviates from the predefined target value. This target value typically represents the ideal outcome you aim to achieve in the optimization process. For example, a GAP value of 0 % indicates that the optimized value exactly matches the target value. A lower GAP value signifies that the solution is closer to the target, indicating a better result. Additionally, the "Time (s)" column represents the time taken to reach this outcome. The specific definition of the target value should be provided in the context of the problem or model.

**Table 5:** Production quantities x(i,t).

	Jan	Feb	Marc
Product1	3	2	2
Product2	1	4	0
Product3	5	0	1
Product4	1	3	0
Product5	1	3	4
Product6	7	0	0
Product7	1	2	0
Product8	2	0	0
Product9	1	3	2
Product10	2	0	3

**Table 6:** Product-Class Assignments q(i,c).

	Class1	Class2	Class3	Class4
Product1	–	1	–	–
Product2	–	1	–	–
Product3	1	–	–	–
Product4	–	–	–	1
Product5	1	–	–	–
Product6	–	1	–	–
Product7	–	–	–	1
Product8	–	1	–	–
Product9	–	1	–	–
Product10	–	–	1	–

**Table 7:** The storage locations z(l,c).

	Class1	Class2	Class3	Class4
Location1	1	–	–	–
Location2	1	–	–	–
Location3	1	–	–	–
Location4	1	–	–	–
Location5	1	–	–	–
Location6	1	–	–	–
Location7	–	1	–	–
Location8	–	1	–	–
Location9	–	1	–	–
Location10	–	1	–	–
Location11	–	1	–	–
Location12	–	1	–	–
Location13	–	1	–	–
Location14	–	1	–	–
Location15	–	1	–	–
Location16	–	1	–	–
Location17	–	1	–	–
Location18	–	1	–	–
Location19	–	1	–	–
Location20	–	1	–	–
Location21	–	–	1	–
Location22	–	–	1	–
Location23	–	–	1	–
Location24	–	–	–	1
Location25	–	–	–	1
Location26	–	–	–	1
Location27	–	–	–	1
Location28	–	–	–	1

**Table 8:** The numerical results of sample problem.

Object Value	GAP [%]	Time (s)
1648,28	0	181,2

After conducting the analysis, it is evident that the sample problem was successfully optimized under the provided constraints, resulting in the best solution. This solution effectively determines production quantities, class assignments, and storage locations based on the provided tables. The key research findings are as follows:

1. The choice to assign equal weights to the criteria in this study was a strategic decision aimed at creating a uniform standard for evaluation. This approach facilitates a direct comparison of the TOPSIS and COI methods under consistent conditions. By doing so, we could objectively assess the strengths of TOPSIS in a multi-criteria decision-making context. The goal was to showcase the adaptability and strength of TOPSIS in diverse situations, irrespective of the individual significance of each criterion. While this equal weighting offers a starting point for analysis, it is by no means a fixed rule. We encourage warehouse managers to modify these weights to reflect the unique demands and priorities of their operations. Such flexibility in weight allocation allows for a more customized and relevant application of the TOPSIS method in the practical realm of warehouse management.
2. Evaluating the performance of various classification methods within multi-criteria storage systems is essential for understanding their effectiveness. In our study, we initially assign equal weight to all criteria to provide a standardized baseline for comparing different methods. This approach enables a fair assessment of each method's capabilities in optimizing warehouse efficiency, costs, and production planning, thereby contributing to well-rounded decision-making processes. However, we recognize the potential benefits of a more tailored weighting scheme.
3. Real-time application and dynamic variable analysis could further refine our proposed mathematical model and decision support system. By adapting the model to real-time data and changing conditions, and potentially varying the weight assigned to each criterion based on strategic importance, our model could yield even more practical and relevant solutions. This would allow the model to more accurately reflect the dynamic priorities of warehouse operations.
4. This approach, with equal weight assigned to the criteria, provides valuable insights for improving class-based production planning and multi-criteria warehouse systems, benefiting food companies and similar industries seeking to optimize their warehouse management processes.

### 4.3 Sensitivity analysis

In this section, we will comprehensively explore the outcomes resulting from our sensitivity analysis, which utilizes both the existing solution and randomly generated C* values, as meticulously laid out in Table-9. The primary aim of this segment is to promote a profound understanding of the profound effects exerted on the decision-making process by C* values and product-class/location assignments.

**Table 9:** Comprehensive examination of C values and product-class/location assignments.

	Current Solution Value			Random Solution Value 1			Random Solution Value 2		
	C* Value	Class	Location	C* Value	Class	Location	C* Value	Class	Location
Product1	0,420	2	7–20	0,601	1	1–8	0,396	3	10–23
Product2	0,488	2	7–20	0,439	2	9–13	0,672	1	1–4
Product3	0,578	1	1–6	0,505	1	1–8	0,461	2	5–9
Product4	0,252	4	24–28	0,468	2	9–13	0,531	1	1–4
Product5	0,686	1	1–6	0,475	2	9–13	0,417	3	10–23
Product6	0,467	2	7–20	0,397	3	14–23	0,394	3	10–23
Product7	0,272	4	24–28	0,382	3	14–23	0,412	3	10–23
Product8	0,452	2	7–20	0,413	3	14–23	0,391	3	10–23
Product9	0,423	2	7–20	0,332	4	24–26	0,335	4	24–28
Product10	0,365	3	21–23	0,468	2	9–13	0,315	4	24–28

#### 4.3.1 The impact of C values and the significance of product-class and location assignments

Our quest for insight begins with Table 9, meticulously designed to elucidate the dynamic relationship between changes in C* values and their repercussions on decision-making. The juxtaposition of existing C* values with their randomly selected counterparts casts a spotlight on the nuanced influences these values yield on our decision outcomes. This comprehensive analysis underscores the remarkable accuracy of high C* values in forecasting outcomes with exceptional precision. It is important to note that our analysis further reveals scenarios in which adopting higher C* values can open the door to more cost-effective solutions. A particularly notable revelation is the 30 % divergence between the existing C* value and the randomly selected C* value 1, highlighting that the effectiveness of our existing solution transcends a rigid fixation on a single C* value.

Furthermore, our examination extends to Table-9, underscoring the pivotal role played by product-class and location assignments in achieving favorable results. The degree of accuracy in these classifications significantly influences the determination of optimal stock levels. It is crucial to note the similarities evident between product-class and location-class assignments in the existing solution and random solutions, emphasizing the substantial impact of strategic planning and operational adaptability.

#### 4.3.2 Percentage and quantity of changes

Building upon our extensive analysis of C* values and assignments, as meticulously unveiled in Table-9, our scholarly journey proceeds to an appraisal of percentage and quantity changes, meticulously chronicled in Table 10. This crucial component of our investigation provides a panoramic understanding of the differentiations between random solutions and the existing solution. The change in C* values emerges as a linchpin in our endeavor to gauge the sensitivity of our results. Furthermore, the shifts in solution times delineate the overarching flexibility in our operational processes and their nimble responsiveness when confronted with scenarios mandating expeditious solutions. The quantities of change underscore the amplitude to which distinct solutions diverge concerning parameters such as cost, time, and other pivotal criteria.

**Table 10:** Summary of sensitivity analysis results.

	Current Solution	Random Solution 1	Random Solution 2	Change Amount 1-2	Change Amount 1-3
Objective Value	1648,28	1596,13	1707,34	52,15	−59,06
GAP (%)	0,00 %	3,15 %	3,81 %	−3,15 %	−3,81 %
Time (s)	181,2	179,8	182,7	1,4	−1,5

#### 4.3.3 Implications of sensitivity analysis

This meticulous sensitivity analysis, manifest in the dual personas of Table 9, Table 10, embodies a potent instrument for the optimization of decisions. It unveils a comprehensive canvas portraying both the strengths and vulnerabilities of the existing solution, thereby laying the groundwork for more informed and judicious decision-making. It is prudent to acknowledge that the results we garner from our analysis are notably subject to the idiosyncrasies and objectives distinctive to each business entity. Therefore, the embrace of a flexible and adaptable approach stands as an imperative hallmark for any organization seeking to chart an empowered course guided by empirical insights.

## 5 Conclusion

In conclusion, this study has explored the integration of the TOPSIS method for multi-criteria storage systems classification and class-based production planning. The results not only underscore the optimization of class-based production planning and product positioning but also highlight its potential for enhancing warehouse efficiency and reducing operational costs. In our model, the utilization of the TOPSIS method enhances the decision-making process by offering a more nuanced evaluation of multiple criteria, crucial for class-based product placement in warehouses. This approach stands out from traditional COI-based methods by providing a more comprehensive analysis, particularly in scenarios involving a diverse range of criteria. The TOPSIS method's ability to effectively process and integrate these varying criteria, even under the assumption of equal weighting, demonstrates its added value in our model. This adaptability makes our approach particularly relevant for complex warehouse environments, where a range of factors must be considered to optimize efficiency and reduce costs. This adaptability and robustness are particularly advantageous for food companies, and the insights gained in this study set a strong precedent for addressing analogous challenges in other industries. However, it is essential to acknowledge the scope of this study's limitations. While this research successfully demonstrated the optimization of class-based production planning and product positioning, it's important to recognize that further exploration is warranted to quantify the precise enhancements in warehouse efficiency and cost reduction. Such additional investigation will undoubtedly augment the significance of the findings. Moreover, addressing potential limitations or identifying areas for improvement in the implementation or usability of the proposed decision support system flow would bolster its practical relevance. In summary, the superiority of the TOPSIS method lies in its flexibility and adaptability to complex decision-making scenarios, and its application in warehouse management holds great promise for enhancing operational efficiency and cost-effectiveness. Nevertheless, ongoing research and a critical evaluation of limitations and improvements are necessary to fully realize its potential.

For future studies, several recommendations are worth considering. First, a comparative analysis of various classification methods can offer valuable insights into their respective performances and their implications for warehouse efficiency, costs, and production planning. Such an approach would contribute to a more comprehensive decision-making process. Second, future research endeavors should focus on real-time data and dynamic variables to adapt the mathematical model and decision support system flow to dynamic conditions. This would result in more realistic and adaptable solutions, making the proposed approaches even more effective and responsive to changing circumstances. By incorporating these suggestions, researchers can significantly advance the field of class-based production planning and multi-criteria warehouse systems, ultimately achieving higher levels of optimization. Addressing the previously mentioned limitations and further exploring the proposed approaches are crucial steps toward gaining a deeper understanding of their implications and broadening their applicability.

The enhanced discussion on the applicability of this research underlines its potential to make substantial contributions not only to the specific domain of warehouse management and production planning but also to various industries grappling with similar challenges. This research serves as a promising step toward the development of practical solutions that can be implemented to optimize efficiency and reduce costs, aligning with the ever-evolving needs of businesses in a competitive market landscape.

## Funding

This research received no external funding.

## Data availability statement

All data used in this study were randomly generated using the Microsoft EXCEL program, and the analysis codes were developed using the GAMS/BARON program (Version 23.5). Data and codes corresponding to this study are available from the corresponding author upon reasonable request. Since the data were generated specifically for this study and are not stored in a public repository, no accession numbers are available.

## CRediT authorship contribution statement

**Mehmet Akif Yerlikaya:** Writing – original draft, Validation, Software, Resources, Project administration, Methodology, Funding acquisition, Formal analysis, Data curation. **Feyzan Arıkan:** Writing – review & editing, Validation, Project administration, Conceptualization.

## Declaration of competing interest

The authors declare the following financial interests/personal relationships which may be considered as potential competing interests:Mehmet Akif reports article publishing charges was provided by Bitlis Eren University. Mehmet Akif reports a relationship with Bitlis Eren University that includes: employment. Mehmet Akif has patent pending to Mehmet Akif.
